# The Nutraceutical Properties of Ovotransferrin and Its Potential Utilization as a Functional Food

**DOI:** 10.3390/nu7115453

**Published:** 2015-11-04

**Authors:** Francesco Giansanti, Loris Leboffe, Francesco Angelucci, Giovanni Antonini

**Affiliations:** 1 Department of Health, Life and Environmental Sciences, University of L’Aquila, L’Aquila I-67100, Italy; francesco.angelucci@univaq.it; 2 Interuniversity Consortium INBB Biostructures and Biosystems National Institute, Rome I-00136, Italy; loris.leboffe@uniroma3.it (L.L.); giovanni.antonini@uniroma3.it (G.A.); 3 Department of Sciences, Roma Tre University, Rome I-00146, Italy

**Keywords:** ovotransferrin, nutraceutical, functional food, antioxidant

## Abstract

Ovotransferrin or conalbumin belong to the transferrin protein family and is endowed with both iron-transfer and protective activities. In addition to its well-known antibacterial properties, ovotransferrin displays other protective roles similar to those already ascertained for the homologous mammalian lactoferrin. These additional functions, in many cases not directly related to iron binding, are also displayed by the peptides derived from partial hydrolysis of ovotransferrin, suggesting a direct relationship between egg consumption and human health.

## 1. Introduction

Ovotransferrin (Otrf) or conalbumin belongs to the family of transferrin iron-binding glycoproteins. In mammals, two different soluble iron-binding glycoproteins are present: (i) serum transferrin, involved in iron transport and delivery to cells and (ii) lactoferrin, involved in the so-called natural immunity. Differently, Otrf is the only soluble glycoprotein of the transferrin protein family present in avian. Otrf is present both in avian plasma and egg white and possesses both iron-transfer and protective properties [1]. Otrf represents about 12%–13% of total egg white proteins and contributes to promoting the growth and development of the chicken embryo mainly preventing the growth of micro-organisms together with other proteins such as lysozyme [2], cystatin [3,4], ovomacroglobulin [5] and avidin [6]. Galliformes (chicken, *Gallus gallus* and turkey, *Meleagris gallopavo*) appear to possess albumens with greater antimicrobial activity than those of the anseriformes (duck, *Anas platyrhynchos*), possibly due to higher concentrations of ovotransferrin and of the broad active c-type lysozyme [7]. However, recent evidence indicates that Otrf is endowed not only with the antibacterial activity related to iron withholding, but also with other roles related to the protection of the growing embryo, including: regulation of iron absorption; immune response; and anti-bacterial, anti-viral and anti-inflammatory properties. Some of these properties are shared by both the human protein homologues and peptides deriving from its partial enzymatic hydrolysis [8], being in this latter case also increased.

The state of the art hereby described suggests that Otrf and its peptides can be used as functional food ingredients and as important components for nutraceuticals, being characterized both by protective functions and by substantial nutritional benefits; for these reasons, the utilization of Otrf and its peptides in functional foods can present several additional advantages over other natural compounds.

## 2. Ovotransferrin Synthesis and Structure

Otrf is a monomeric glycoprotein containing 686 amino acids, with a molecular weight of 77.9 kDa and an isoelectric point of 6.0 [9,10].

The avian transferrin gene is transcripted in the liver and the oviduct. In the liver, the transferrin is secreted in the serum, where it is involved in iron transport and storage, while the oviduct transferrin (ovotransferrin) is secreted at high levels in the egg white. In particular, progesterone and oestrogen regulate the expression of Otrf in the oviduct: oestrogen can interact with chromatin through a nuclear receptor protein stimulating transcription and synthesis of the protein precursor [11]. Instead, the transcription of serum Otrf may depend on iron concentration [12]. Although the serum transferrin and Otrf have the same amino acidic sequence, they differ in the glycosylation sites. The glycan of Otrf is constituted by four residues of mannose and four residues of *N*-acetylglucosamine whereas serum transferrin is composed of two residues of mannose, two residues of galactose, three residues of *N*-acetylglucosamine, and one or two residues of sialic acid at its C-terminus [13,14,15].

**Figure 1:**
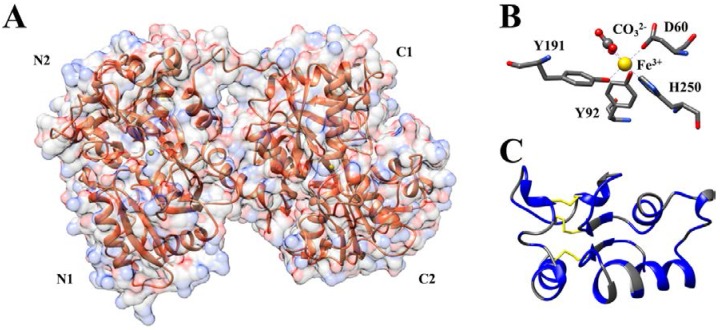
(**A**) Ribbon representation and the solvent-accessible surface (in transparency) of holo-Otrf (PDB ID:1OVT) [16]. N1, N2 and C1, C2 indicate the subdomains of each lobe. (**B**) The N-lobe iron binding site of hen’s ovotransferrin (PDB ID:1OVT). The amino acids involved in iron binding are shown in sticks. H-bonds are displayed by purple broken lines. Both in (**A**) and (**B**), the iron is indicated as a yellow sphere. (**C**) Schematic ribbon representation of the OTAP-92 peptide. The three disulfide linkages are represented in yellow, while the hydrophobic residues are highlighted in blue. The peptide is shown with the same conformation displayed in the intact protein. Molecular graphic images were produced using the UCSF chimera package [20].

Like the mammalian transferrins, the single chain of Otrf consists of two globular lobes (N- and C-lobes), interconnected by a α-helix of nine amino acidic residues (residues 333–341) that can be released by tryptic digestion. Each lobe contains an iron binding site and is divided in two domains, (domains N1 and N2 in the N-lobe and domains C1 and C2 in the C-lobe, respectively; Figure 1A) [16,17,18].

As shown in Figure 1, the domains are linked by anti-paralleled β-strands. The N- and C-lobes show about 38% sequence homology; indeed, it is has been hypothesized that all members of transferrin family resulted from gene fusion and duplication [19]. Fifteen disulfide bridges stabilize the structure. Six of them are conserved in both lobes, while three are present only in the C-lobe, conferring to the lobes a different metal affinity.

Each lobe has the capability to reversibly bind one Fe^3+^ ion along with one CO_3_^2−^ anion. Although each lobe displays a high sequence homology, they show different iron-binding properties. In particular, the (approximate) iron binding affinity is 1.5 × 10^18^ M^−1^ for the C-lobe and 1.5 × 10^14^ M^−1^ for the N-lobe. As mentioned before, this difference is due to the presence, in the C-lobe, of an extra interdomain disulfide bond, Cys478-Cys671, which confers less flexibility and possibly less affinity towards Fe^3+^ ion [21].

From a stereochemical point of view, the iron binding pocket is conserved among all members of transferrins [16,17,18,22,23,24]. As shown in Figure 1B, in the N-lobe there are two phenolate oxygens of two tyrosine residues (Tyr92 and Tyr191), a carboxylate oxygen of the aspartic acid (Asp60), and the imidazole nitrogen of the histidine (His250), together with two oxygens of the synergistically bound CO_3_^2−^ anion. In C-lobe, the corresponding amino acids are Asp 395, Tyr524, Tyr431 and His592, respectively. In this latter, Arg460 keeps in place the carbonate moiety while in the N-lobe Arg121 plays the same role. Upon iron binding, each lobe of Otrf undergoes large conformational change; the observed movement is necessary to bury the metal inside the polypeptide chain given that, in its absence, the iron-coordinating amino acidic residues are solvent exposed.

## 3. Antibacterial Activity of Ovotransferrin and Its Peptides

Among the several protective functions of Otrf, the most important one is likely to be the antibacterial activity, which is directly related to the Otrf’s ability to bind iron (Fe^3+^), making it unavailable for bacterial growth [25,26]. This bacteriostatic activity is reversed by adding iron ions to the medium and it is blocked by iron saturation [27]; moreover, it can be enhanced by (i) adding carbonate ion [28] which is one of the iron ligands in the Otrf metal binding site [29]; (ii) increasing the pH from 6 to 8 [27]; (iii) and immobilizing Otrf by covalent linkage to Sepharose 4B [30]. An increase of the bacteriostatic activity towards *E. coli* O157:H7 as iron chelator was demonstrated using a combination of ovotransferrin, NaHCO_3_, and EDTA [31]. The antibacterial activity was demonstrated also in an *in vivo* study using newborn guinea pigs orally infected with *E. coli* 0111 B4 [32]. On the contrary, citrate exerts an antagonistic effect in those bacteria that possess a receptor for iron-citrate complex [32]. The most sensitive species to the iron deprivation effect of Otrf are *Pseudomonas* spp., *Escherichia coli*, *Streptococcus mutans*, while the most resistant ones are *Proteus* spp., and *Klebsiella* spp. [32], according to the ability of these latter bacteria to produce molecules (*i.e.*, siderophores) able to compete with Otrf for iron binding. However, it is worth noting that some bacterial species that are also human pathogens, e.g., *Neisseria meningitidis*, *Neisseria gonorrhoeae* and *Moraxella catarrhalis*, have developed a mechanism for acquiring iron directly from transferrin-like proteins through surface receptors capable of specifically binding ovotransferrin [33].

Other studies suggested that part of the antibacterial activity of Otrf is not simply due to the removal of iron from the medium, but also involves more complex mechanisms related to a direct binding of Otrf to the bacterial surface. As a matter of fact, it has been initially demonstrated that the antibacterial activity of Otrf decreased when the protein is separated by dialysis from the bacteria, a condition in which it can exert only the iron-chelating property [34]. Accordingly, it was shown that Otrf is able to permeate the *E. coli* outer membrane and access the inner membrane, causing both ion leakage inside bacteria and the uncoupling of the respiration-dependent energy production [35]. The antibacterial effect of Otrf towards *Salmonella enterica* (*serovar Choleraesuis*) has been also demonstrated to be dependent on culture conditions that either favor or hinder binding Otrf to the bacterial surface [36]. These data suggest that this Otrf function, not related to iron binding, could be due to a cationic bactericidal domain which, as other transferrins, is located in the N-lobe [35].

The isolation of the bactericidal domain of Otrf, was carried out by Zhou and Smith in 1990 by a partial acid proteolysis. OTAP-92 is a peptide of 9.9 kDa, consisting of 92 aminoacidic residues (Leu109-Asp200) showing strong sequence similarity with defensins [37]. This peptide is characterized by three disulfide bridges (Cys115-Cys197, Cys160-174, and Cys171-Cys182) and several positively charged residues (Figure 1, panel C).

It has been suggested that the antibacterial action of OTAP-92 may be due to its relatively high alkalinity and to the cysteine array. Both these features are shared by native antibacterial peptides [38,39] and by insect defensins whose mechanism of action involves the blocking of the voltage-dependent K^+^ channels [38,39,40,41,42].

As concerning the possible biotechnological applications of the Otrf antibacterial activity, Ko and coworkers [31] showed that a combination of Otrf, NaHCO_3_, EDTA and/or Lysozyme have a potential growth inhibition effect against *E. coli* O157:H7 or *L. monocytogenes*, demonstrating also a potential application of Otrf as a natural preservative for food (*i.e.*, pork chops and commercial hams) [31,43,44].

## 4. Antiviral Activity of Ovotransferrin and Its Peptides

The antiviral activity of Otrf was firstly demonstrated towards the avian herpesvirus Marek’s disease virus (MDV), and no correlation between antiviral efficacy and iron saturation was found [45]. It has been postulated that the ovotransferrin antiviral activity towards MDV is associated with two Otrf fragments: DQKDEYELL (hOtrf219-27) and KDLLFK (hOtrf269-301 and hOtrf633-638) capable of blocking Marek’s disease virus infection in chicken embryo fibroblasts (CEF), even though the infection blocking efficiency of the isolated peptides is lower than that of the intact protein [46]. Interestingly, from an evolutionary point of view, these two Otrf peptides share sequence homology with two protein fragments, derived from human and bovine lactoferrin, known to be effective against *Herpes simplex* Virus (HSV-1) [47].

## 5. Antioxidant Activity of Ovotransferrin and Its Peptides

Ovotransferrin is a superoxide dismutase-mimicking protein exhibiting a superoxide radical (O_2_^•−^)-scavenging activity. Furthermore, self-cleaved Otrf exhibited O_2_^•−^ scavenging capacity greater than intact protein [48,49]. Accordingly, it has been shown that, after digestion by thermolysin and pepsin, the resulting Otrf hydrolysates possessed significantly higher oxygen radical absorption capacity (1.69 μmol Trolox equivalent mg^−1^) than natural ovotransferrin [50].

Kim and coworkers [51] demonstrated that the antioxidant effects of hen’s ovotransferrin and of its hydrolyzed peptides is approximately 3.2–13.5 times higher than superoxide anion scavenging activity than Otrf, with the maximum activity displayed by octapeptides. Similar results were obtained for oxygen radical absorbance capacity assay and against the oxidative stress-induced DNA damage in human leukocytes [51]. In addition, Otrf-derived peptides showed synergistic antioxidant effects with Vitamin C, epigallocatechin gallate (EGCG), and caffeic acid [52]. Otrf hydrolyzate (obtained using enzymes such as protamex, alkalase, trypsin, and α-chymotrypsin) showed protective effects against oxidative stress including DNA damage in human leukocytes [53].

The conjugation of ovotransferrin with catechin (a polyphenol antioxidant found in tea, wine, fruits, with high affinity to bind protein) improved the oxygen radical absorbance capacity of the protein. The ovotransferrin-catechin conjugates were prepared using a hydrogen peroxide–ascorbic acid pair as radical initiator system and alkaline method [54]. Moreover, it has been also shown that catechin, after the conjugation reaction and after UPLC (Ultra-Performance Liquid Chromatography), MALDI-TOF (Matrix Assisted Laser Desorption Ionization Time-of-Flight) and Liquid chromatography-tandem mass spectrometry (LC-MS-MS) analysis, was bound to lysine (residues 327) of the Otrf peptide DLLFKDSAIMLK (residues 316–327) and to glutamic acid (residues 186) of the Otrf peptide FFSASCVPGATIE (residues 174–186) present in ovotransferrin N-lobe [54].

Autocleaved Otrf was shown to (i) hinder effectively the discoloration of ß-carotene (used as radical target in a bleaching test); (ii) prevent the oxidation of linoleic acid during five days of storage at 4 °C; and (iii) show strong Cu^2+^- and Ca^2+^-binding capacities, suggesting that it could be a good source of natural antioxidants. Once again, its metal-chelating activity could be at least partly responsible for the observed antioxidant mechanisms [55].

In conclusion, the use of Otrf-derived conjugates as a novel proteinaceous antioxidants as ingredient in the nutraceutical and functional food is desirable.

## 6. Anti-Inflammatory Activities of Ovotransferrin and Its Peptides

During inflammation in avians, Otrf, as well as the positive acute phase protein (APP), is up-regulated both *in vivo* and *in vitro* [56,57,58,59,60,61,62,63]; its levels in blood remain elevated as long as the inflammation persists [64,65]. Indeed, the use of blood Otrf concentration as an infection and inflammation marker in chickens has been hypothesized [66]. Otrf may act directly as an immunomodulator [63], even though its role in inflammation preventing microbial growth [67] and acting as an antioxidant against Fenton reaction products [36] cannot be ruled out.

More recently, two tripeptides, IRW and IQW, both derived from Otrf hydrolysis, were found to attenuate TNF-α-induced inflammatory responses and oxidative stress in vascular endothelial cells [68]. Furthermore, other peptides derived from ovomucoid showed an immunomodulating activity against T-cells and macrophage-stimulating activities *in vitro* [69], indicating that they also can be good candidates for pharmaceutical use in humans [70].

## 7. Other Protective Activities of Ovotransferrin and Its Peptides

In the last years, it has been reported that Otrf underwent thiol-linked auto-cleavage after reduction, and produced partially hydrolyzed products with very strong anticancer effects against colon (HCT-116) and breast cancer (MCF-7) by inducting apoptosis [71].

An antihypertensive subsidiary function of the Otrf was detected in the hen Otrf’s peptide KVREGT, showing an IC_50_ value of 9.1 μM towards angiotensin Ι-converting enzyme [72]. Moreover, the same peptide displayed both a strong ACE-inhibitory and a vasodilatory activities [73].

Ovotransferrin has also shown both *in vitro* and *in vivo* development promoting activity, which has been associated to its iron binding/transport capabilities. Ovotransferrin is transiently expressed and secreted in large amounts during the *in vitro* differentiation of hypertrophic chondrocytes into osteoblast-like cells. Cells expressing ovotransferrin also co-express ovotransferrin receptors, suggesting a self-regulatory mechanism in the control of chondrocyte differentiation to osteoblast-like cells [74,75,76,77,78,79].

Otrf shares with human and bovine lactoferrin a proteolytic activity catalyzing the hydrolysis of several synthetic substrates [80]. Serine protease inhibitors PMSF (phenylmethylsulfonyl fluoride), LPS (lipopolysaccharide) and Pefabloc impair this proteolytic activity suggesting that it is similar to that of serine proteases [81,82]. This function is conserved in several mammalian lactoferrins but not in serum transferrins, and thus it is plausible that it belongs to the protective functions of Otrf, although the physiological target has not been identified, yet.

Lastly, Ibrahim *et al.* [83] demonstrated the efficiency of Otrf to serve as a drug carrier to improve the solubility of three water-insoluble antibiotics and to facilitate their specific delivery into microbial or infected cells. For a complete overview of the Otrf properties, see Table 1.

**Table 1:** Physiological and pharmacological activities of Ovotransferrin (Otrf) and its peptides identified to-date.

PROTEIN OR PEPTIDE	ACTIVITY	MECHANISM/S	REFERENCES
Ovotransferrin (whole molecule)	ANTIMICROBIAL		
	BACTERIAL SENSITIVITY (BACTERIOSTATIC)	Otrf iron Binding (Iron withholding)	[25,27,30,32]
	BACTERIAL SENSITIVITY (BACTERICIDAL)	Membrane damage	[7,25,32]
	BACTERIAL RESISTANCE	Bacterial production of Iron chelators or Tbp1 and 2	[33]
	ANTIBACTERIAL (FOOD PRESERVATIVE)	In combination with EDTA and/or Lysozyme prevents *E. coli* O157:H7 or *L. monocytogenes*, proliferation	[43]
	ANTIVIRAL	Viral adsorption inhibition	[45]
	ANTIOXIDANT	Iron Binding SuperOxide Dismutase (SOD)-like activity Fenton’s reaction inhibition Catechin conjugation	[49,50]
	FLOGOSIS MARKER	Otrf Belongs to Acute Phase Proteins (APP). Recognition and protection against invading pathogens, and restoration of the physiological homeostasis. Otrf upregulation of interleukin-6, nitric oxide, and matrix metalloproteinase.	[61,63,64,66]
	IMMUNE/ANTI-INFLAMMATION	Modulation of macrophages and heterophils functions. SOD-Like Activity	[49,63,64]
	PROTEOLYTIC	Hydrolysis of Haemophilus colonization factors and of several synthetic substrates	[80]
	GROWTH FACTOR:		
	CARTILAGE NEOVASCULARIZATION	Chemotactic factor for endothelial cells. Iron delivery	[59]
	CHONDROGENESIS AND OSTEOGNESIS REGULATION	Iron delivery	[77,78]
	MYOTROPHIC	Iron delivery	[74]
	NEUROTROPHYC	Iron delivery	[79]
	CARRIER FOR DRUG DELIVERY	Binding and delivery of water insoluble antibiotics (sulphantibiotics)	[83]
Otrf peptide OTAP-92	ANTIMICROBIAL	Bacterial Membrane damage	[41]
Otrf peptides219–227; 269–301; 633–638	ANTIVIRAL	Viral adsorption inhibition	[46]
Reduced autocleaved Otrf (rac-Otrf)	ANTICANCER/ANTIPROLIFERATION	Apoptosis induction	[71]
	ANTIOXIDANT	Autocleaved Otrf as preservative to prevent ß-carotene discoloration	[55]
Otrf Peptides (IRW or IQW)	ANTINFLAMMATORY	Attenuate TNF-α-induced inflammatory responses	[68]
Otrf peptide (KVREGT)	ANTIHYPERTENSIVE	Inhibition of Angiotensin Ι-Converting Enzyme.	[72,73]
Otrf Peptides (DLLFKDSAIMLK) (FFSASCVPGATIE)	ANTIOXIDANT	Catechin conjugation	[54]
Otrf peptides (mix obtained using: protamex, or alkalase, or trypsin, or α-chymotrypsin)	ANTIOXIDANT	Synergistic antioxidant effects with vitamin C, epigallocatechin gallate (EGCG), and caffeic acid.	[52,53]

## 8. Conclusions

Many of the Otrf’s protective properties, described in this review and outlined in Table 1, contribute, in addition to the well-known iron transfer activity, to the proper development of the chicken embryo. Moreover, the demonstration that several defensive properties of Otrf are also possessed by its proteolytic fragments and that these properties may be of importance for human wellness strongly support the use of egg white (preferably raw or cooked at low temperature to preserve ovotransferrin properties) and its derivatives as dietary additives in normal and pathological human conditions.
